# Neural Signals Related to Outcome Evaluation Are Stronger in CA1 than CA3

**DOI:** 10.3389/fncir.2017.00040

**Published:** 2017-06-07

**Authors:** Sung-Hyun Lee, Namjung Huh, Jong Won Lee, Jeong-Wook Ghim, Inah Lee, Min W. Jung

**Affiliations:** ^1^Center for Synaptic Brain Dysfunctions, Institute for Basic ScienceDaejeon, South Korea; ^2^Neuroscience Graduate Program, Ajou University School of MedicineSuwon, South Korea; ^3^Department of Brain and Cognitive Science, Seoul National UniversitySeoul, South Korea; ^4^Department of Biological Sciences, Korea Advanced Institute of Science and TechnologyDaejeon, South Korea

**Keywords:** hippocampus, reinforcement learning, chosen value, dynamic foraging task, decision making, T-maze, rat

## Abstract

We have shown previously that CA1 conveys significant neural signals necessary to update value of the chosen target, namely chosen value and reward signals. To better understand hippocampal neural processes related to valuation, we compared chosen value- and reward-related neural activity between the CA3 and CA1 regions. Single units were recorded with tetrodes from the dorsal CA3 and CA1 regions of rats performing a dynamic foraging task, and chosen value- and reward-related neural activity was estimated using a reinforcement learning model and multiple regression analyses. Neural signals for chosen value and reward converged in both CA3 and CA1 when a trial outcome was revealed. However, these neural signals were stronger in CA1 than CA3. Consequently, neural signals for reward prediction error and updated chosen value were stronger in CA1 than CA3. Together with our previous finding that CA1 conveys stronger value signals than the subiculum, our results raise the possibility that CA1 might play a particularly important role among hippocampal subregions in evaluating experienced events.

## Introduction

As a structure known for its essential role in encoding episodic memory, the hippocampus has not been a popular target for investigation of the neural underpinning of value-based decision-making. However, when we remember a past episode, we usually remember not only what happened, but also its affective component—how good or bad—as well. In this regard, significant value signals have been found in the human (Tanaka et al., 2004; Bornstein and Daw, 2013) and rat (Lee et al., 2012) hippocampus, raising the possibility that factual and value information may be conjunctively encoded in the hippocampus such that the memory of an event is inseparable from the memory of its value. If so, recalling factual information will automatically evoke associated value information, which would be useful for making advantageous choices in the future when one encounters a similar situation as experienced before (Wimmer and Shohamy, 2012). Concurrent coding of factual and value information would be also useful for simulating hypothetical outcomes and assessing their values. It is now well-established that the hippocampus is important not only for memory, but also for imagining new experiences (Buckner, 2010; Schacter et al., 2012; Gaesser et al., 2013; Mullally and Maguire, 2014). Value information represented in the hippocampus would be useful for simulating most probable and rewarding scenarios for maximizing value.

Currently, hippocampal neural processes related to value-based decision making are poorly understood. We have shown previously that CA1 conveys strong and robust value signals, whereas value signals are only weak in its neighboring structure, subiculum, in rats (Lee et al., 2012). An important question then is whether and how the other hippocampal subregions process value-related information. A particularly important question is characteristics of value-related neural signals in CA3, which provides the heaviest afferent projections to CA1 (Amaral et al., 1990). In the present study, to better understand hippocampal neural processes related to updating values of experienced events, we compared value-related neural activity between CA3 and CA1. We found that chosen value and reward signals are stronger in CA1 than CA3. Our results argue against the possibility that CA3 is the major source of value signals found in CA1. They also suggest that CA1 may play a particularly important role among hippocampal subregions in evaluating experienced events.

## Materials and methods

### Animals

Four young (9 weeks old, 300–350 g) male Sprague-Dawley rats were individually housed in their home cages and initially allowed free access to food and water with extensive handling for 1 week. They were then gradually water deprived so that their body weights were maintained at 80–85% of their free-feeding weights throughout the experiments. The experiments were performed in the dark phase of a 12 h light/dark cycle. The experimental protocol was approved by the Ethics Review Committee for Animal Experimentation of Korea Advanced Institute of Science and Technology.

### Behavioral task

**Figure 1:**
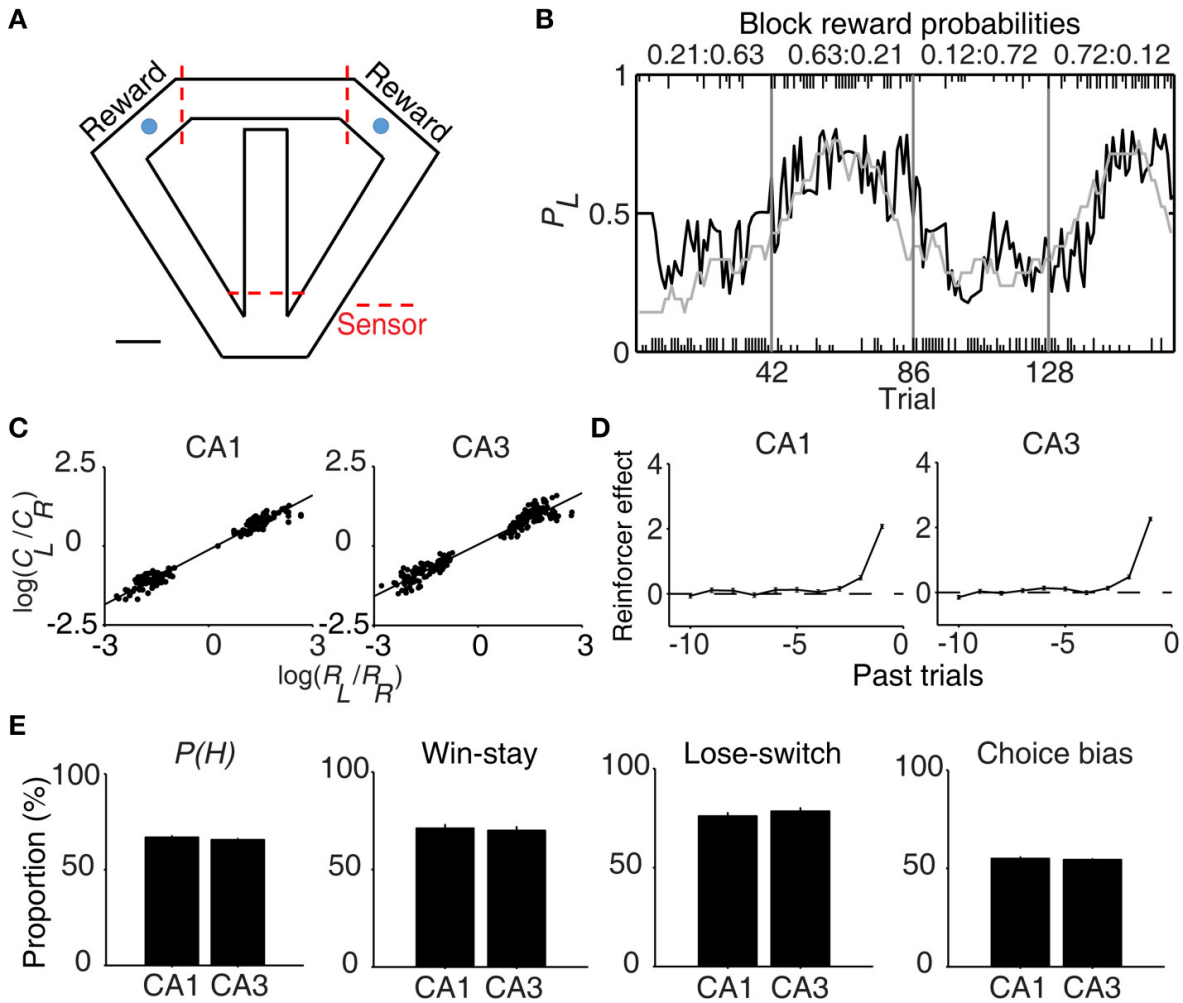
Behavioral performance. **(A)** The modified T-maze used for rats. The rats were allowed to choose freely between two targets (blue circles) that delivered water in a probabilistic manner. Breaking a photobeam sensor in front of a reward site (the two red dashed lines on top) triggered an auditory tone (CS, 1 s) that signaled a trial outcome. Water reward was delivered at the CS offset in rewarded trials. Calibration, 10 cm. **(B)** Rat's choice behavior during one example session. The probability of choosing the left target (*P*_*L*_) is plotted in moving average of 10 trials (gray curve). The black curve represents choice probability predicted by an RL model. Tick marks denote trial-by-trial choices of the rat (upper, left choice; lower, right choice; long, rewarded trial; short, unrewarded trial). Each session consisted of four blocks of trials with different combinations of reward probabilities. Vertical lines denote block transitions and numbers on top indicate reward probabilities used in this example session. **(C–E)** Comparison of choice behavior during CA1 and CA3 recording sessions. **(C)** The relationship between log choice ratio (ordinate) and log reinforcement ratio (abscissa) is shown separately for CA1 and CA3 recording sessions. Each data point was obtained by analyzing steady-state behavioral data (trials after the proportion of higher-reward-probability target choices reaching >90% of the maximum value in each block in 7-trial moving average) during one block of trials. **(D)** Effects of past rewards on the rat's choice are shown separately for CA1 and CA3 recording sessions. The influence of past rewards on the rat's choice was estimated using a logistic regression model. Shown are regression coefficients averaged across four rats (mean ± SEM). Positive coefficients indicate the animal's tendency to make the same choice that was rewarded in recent trials. **(E)** The proportion of higher-reward-probability target choices [*P(H)*] in each block, the proportion of win-stay (repeating the rewarded choice) in each session, the proportion of lose-switch (switching from unrewarded choice) in each session, and choice bias (the proportion of choosing one target over the other in each session) were compared between CA1 and CA3 recording sessions. No significant difference was found in any of these measures (*t*-test, *p* > 0.1).

The rats were trained in a dynamic foraging task in a modified T-maze as described previously (Lee et al., 2012). The maze (65 × 60 cm, width of track: 8 cm, 3 cm high walls along the entire track except the central bridge; elevated 30 cm from the floor) contained three photobeam sensors to monitor the animal's position in the maze (Figure 1A). The animals were required to navigate from the central stem to either goal site to obtain water reward and come back to the central stem via the lateral alley in each trial. A 2 s delay was imposed at the beginning of each trial by raising the distal portion of the central stem. A fixed amount of water reward (40 μl) was delivered according to a concurrent variable-ratio/variable-ratio reinforcement schedule so that each choice contributed to the ratio requirement of both goals. If water was delivered at the unvisited goal, it remained available in the subsequent trials without additional water delivery until the animal visited there [“dual assignment with hold” (DAWH) task] (Lau and Glimcher, 2005; Huh et al., 2009). The animal's arrival at a goal was detected by a photobeam sensor (placed 6 cm ahead of the water delivery nozzle) and triggered an auditory tone (conditional stimulus or CS, 9 and 1 KHz for rewarded and unrewarded trials) for 1 s, which marked the onset of the reward period. Water was delivered at the CS offset in rewarded trials. The animals performed four blocks of trials in each recording session. The number of trials in each block was 35 plus a random number drawn from a geometric mean of 5 with the maximum of 45 (41.7 ± 1.4 trials per block and 167.0 ± 1.4 trials per session; mean ± *SD*). Reward probability of a goal was constant within a block of trials, but changed across blocks without any sensory cues, so that changes in the probabilities of reward could be discovered only by trial and error. The following four combinations of reward probabilities were used in each session: 0.72:0.12, 0.63:0.21, 0.21:0.63, and 0.12:0.72. The sequence was determined randomly with the constraint that the richer alternative always changed its location at the beginning of a new block.

### Unit recording

**Figure 2:**
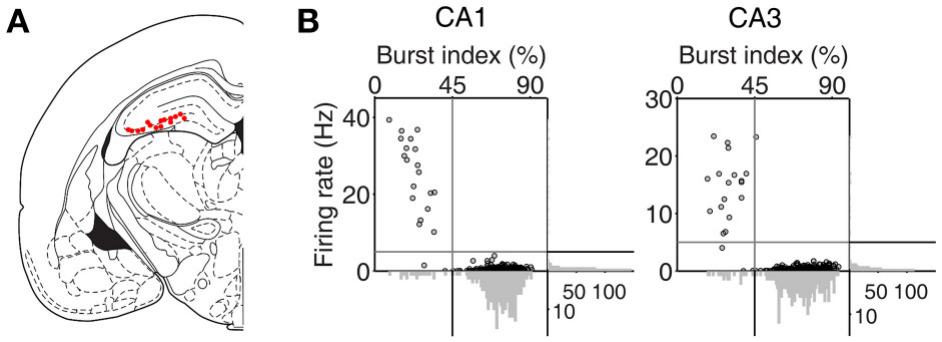
Recording locations and unit classification. **(A)** Single units were recorded first from CA1 and then CA3/DG regions of the rat dorsal hippocampus. Red dots indicate the final recording locations of all tetrodes. Modified from Paxinos and Watson (1998) with permission. **(B)** Unit classification. The recorded units were classified into putative pyramidal cells and putative interneurons based on mean discharge rates and the distribution of inter-spike intervals. Those units with mean discharge rates <5 Hz and the burst index (the percentage of inter-spike intervals shorter than one-fourth of each neuron's mean inter-spike interval) >45% were classified as putative pyramidal cells and the rest were classified as putative interneurons.

An array of 12, 15, or 24 tetrodes was implanted above the right hippocampus (3.6 mm posterior and 2.2 mm lateral to bregma; 1.5 mm ventral to brain surface) of well-trained (20–30 days of training in the DAWH task before surgery) rats under isoflurane (1.5–2.0% [vol/vol] in 100% oxygen) anesthesia. Following 7 days of recovery from surgery, the rats were further trained in the DAWH task for 7–10 days while tetrodes were gradually advanced toward the CA1 cell body layer. Unit signals were recorded first in the CA1 cell body layer (12–16 sessions) and then in the CA3 cell body layer (9–15 sessions), with 12–15 days of tetrode advancements between two bouts of unit recordings. Some tetrodes passed through the dentate granule cell layer instead of the CA3 pyramidal cell layer. Units recorded in the DG were not analyzed because the number of recorded units was relatively small. Unit signals were amplified with the gain of 10,000, filtered between 0.6 and 6 KHz, digitized at 32 KHz and stored on a personal computer using a Cheetah data acquisition system (Neuralynx; Bozemann, MT, USA). Unit signals were also recorded with the animals placed on the pedestal before and after each experimental session to examine the stability of recorded unit signals. Local field potentials (LFPs) were also recorded from each tetrode (gain, 1,000; band-pass filtering, 0.1–1,000 Hz; digitization, 2 KHz). The head position of the animal was recorded at 30 Hz by tracking an array of light-emitting diodes mounted on the headstage. When CA3 or DG recordings were completed, small marking lesions were made by passing an electrolytic current (30 μA, 20 s, cathodal) through one channel of each tetrode and electrode tracks and marking lesions were verified histologically according to a standard procedure (Baeg et al., 2001). Recoding locations were determined based on the history of electrode advancements and histologically-confirmed electrode tracks and lesion sites (Figure 2A).

### Analysis of behavior

#### Logistic regression analysis

Effects of previous choices and their outcomes on animal's goal choice were estimated using the following logistic regression model (Lau and Glimcher, 2005; Kim et al., 2009):

(1) $$\begin{array}{l} \log\left(\frac{p_{L}(i)}{p_{R}(i)}\right)=\sum_{j=1}^{10}r_{j}^{r}\left(R_{L}\left(i-j\right)-R_{R}\left(i-j\right)\right)\\ \;+\sum_{j=1}^{10}r_{j}^{c}\left(C_{L}\left(i-j\right)-C_{R}\left(i-j\right)\right)+r_{0}, \end{array}$$

where *p*_*L*_(*i*) [or *p*_*R*_(*i*)] is the probability of selecting the left (or right) goal in the *i*-th trial. The variables *R*_*L*_(*i*) [or *R*_*R*_(*i*)] and *C*_*L*_(*i*) [or *C*_*R*_(*i*)] are reward delivery at the left (or right) goal (0 or 1) and the left (or right) goal choice (0 or 1) in the *i*-th trial, respectively. The coefficients $r_{j}^{r}$ and $r_{j}^{c}$ denote the effect of past rewards and choices, respectively, and *r*_0_ is a bias term.

#### Matching law

Steady-state behavioral data was analyzed to test their conformity to the generalized matching law (Baum, 1974) as follows:

(2) $$\frac{C_{L}}{C_{R}}=b{\left(\frac{R_{L}}{R_{R}}\right)}^{a},$$

where *C*_*L*_ (or *C*_*R*_) and *R*_*L*_ (or *R*_*R*_) are choice frequency and reinforcement frequency for the left (or right) goal, respectively. The coefficients *a* and *b* are the sensitivity to the reinforcement ratio and a bias term, respectively.

#### Reinforcement learning (RL) model

Value-related activity of CA3 and CA1 neurons was examined using an RL model. We have shown previously that the “stack probability” (SP) model explains rat's choice behavior in the DAWH task better than a simple *Q*-learning model in terms of Akaike's and Bayesian information criteria (AIC and BIC, respectively; Huh et al., 2009), which was confirmed in the present study (Q-learning model, AIC per trial, 1.211 ± 0.009; BIC per trial, 1.213 ± 0.009; SP model, AIC per trial, 1.181 ± 0.012; BIC per trial, 1.183 ± 0.012, mean ± SEM). The SP model is similar to the simple *Q*-learning model except that values were computed considering that reward probability of the unchosen target increases as a function of the number of consecutive alternative choices. Action selection in the model was based on the softmax action selection rule, in which choice probability varied as a graded function of the difference in action values. Details of the SP model are described in our previous study (Huh et al., 2009).

### Analysis of neural data

#### Unit isolation classification

Putative single units were isolated off-line by manual cluster cutting of various spike waveform parameters using the MClust software (A. D. Redish). Only those clusters with L-ratio <0.15 (0.04 ± 0.00, *n* = 535), and isolation distance > 15 (52.5 ± 3.1; Schmitzer-Torbert et al., 2005) were included in the analysis. Units recorded from CA1 and CA3 were classified into putative pyramidal cells (complex spike cells) and putative inhibitory interneurons (theta cells) based on mean discharge rate and a burst index (the percentage of inter-spike intervals shorter than one-fourth of each neuron's mean inter-spike interval). Those units with mean discharge <5 Hz and the burst index > 45% were classified as putative pyramidal cells, that were included in the analysis, and the rest were classified as putative interneurons (Figure 2B). The majority of classified units were putative pyramidal cells (CA1, 262 of 283, 92.6%; CA3, 231 of 252, 91.7%). Their mean discharge rates and burst index were 0.50 ± 0.49 Hz and 73.3 ± 8.3%, respectively, in CA1 and 0.38 ± 0.34 Hz and 72.3 ± 10.4% (mean ± *SD*), respectively, in CA3.

#### Multiple regression analysis

Neural activity related to the animal's choice and its outcome (i.e., reward) was examined using the following regression model:

(3) $$\begin{array}{l} S\left(t\right)=a_{0}+\sum_{n=0}^{2}[a_{3n+1}C\left(t-n\right)+a_{3n+2}R\left(t-n\right)\\ \;+a_{3n+3}X\left(t-n\right)]+a_{10}L\left(t\right)+a_{11}Y\left(t\right)\\ \;+a_{12}M\left(t\right)+A\left(t\right)+\varepsilon (t), \end{array}$$

where *S*(*t*) is neural firing rate, *C*(*t*), *R*(*t*), and *X*(*t*) indicate the animal's choice, its outcome (or reward), and their interaction in trial *t*, *L*(*t*) is the animal's lateral position (lateral deviation from the midline of the maze), *Y*(*t*) is the Y-position of the animal, *M*(*t*) is the animal's movement speed, ε(*t*) is the error term, and *a*_0_ − *a*_12_ are regression coefficients. *A*(*t*) is a set of autocorrelation terms (neural firing rates during the same analysis time window in the previous five trials):

$$A\left(t\right)=\sum_{n=1}^{5}a_{n+12}S(t-n),$$

where *a*_13_ − *a*_17_ are regression coefficients.

Value-related neural activity was examined using the following regression model:

(4) $$\begin{array}{l} S\left(t\right)\;=\;a_{0}+a_{1}C\left(t\right)+a_{2}R\left(t\right)+a_{3}X\left(t\right)+a_{4}Q_{L}\left(t\right)+a_{5}Q_{R}\left(t\right)\\ \;+a_{6}Q_{c}\left(t\right)+a_{7}L\left(t\right)+a_{8}Y\left(t\right)\\ \;+a_{9}M\left(t\right)+A\left(t\right)+\varepsilon \;(t), \end{array}$$

where *Q*_*L*_(*t*) and *Q*_*R*_(*t*) indicate the action values for the leftward and rightward goal choices in trial *t*, respectively, that were estimated with the SP model, and *Q*_*c*_(*t*) denotes the chosen value (value of the chosen target in each trial).

Neural activity related to reward prediction error (RPE) and updated chosen value (*upQ*_*c*_) was examined using the following regression models:

(5) $$\begin{array}{l} S\left(t\right)=a_{0}+\sum_{n=0}^{2}[a_{3n+1}C\left(t-n\right)+a_{3n+2}R\left(t-n\right)\\ \;+a_{3n+3}X\left(t-n\right)]+a_{10}L\left(t\right)+a_{11}Y\left(t\right)\\ \;+a_{12}M\left(t\right)+A\left(t\right)+\varepsilon (t), \end{array}$$

(6) $$\begin{array}{l} S\left(t\right)\;=\;a_{0}+a_{1}C\left(t\right)+a_{2}R\left(t\right)+a_{3}X\left(t\right)+a_{4}Q_{L}\left(t\right)+a_{5}Q_{R}\left(t\right)\\ \;+a_{6}Q_{c}\left(t\right)+a_{7}L\left(t\right)+a_{8}Y\left(t\right)\\ \;+a_{9}M\left(t\right)+A\left(t\right)+\varepsilon \;(t), \end{array}$$

where *RPE* = *R*(*t*) − *Q*_*c*_ (*t*) and *upQ*_*c*_(*t*) = *Q*_*c*_(*t*) + α*RPE*. The parameter α is the learning rate of the SP model that was estimated for each rat using a maximum likelihood procedure (Sul et al., 2010).

The following regression model was used to analyze RPE- and updated chosen value-related neural activity at each reward site separately:

(7) $$\begin{array}{l} S\left(t\right)=a_{0}+a_{1}C\left(t\right)+a_{2}Q_{L}\left(t\right)+a_{3}Q_{R}\left(t\right)+a_{4}upQ_{c}(t)\\ \;+a_{5}L\left(t\right)+a_{6}Y\left(t\right)+a_{7}M\left(t\right)+A\left(t\right)+\varepsilon \;(t), \end{array}$$

(8) $$\begin{array}{l} S\left(t\right)=a_{0}+a_{1}Q_{L}\left(t\right)+a_{2}Q_{R}\left(t\right)+a_{3}RPE+a_{4}L\left(t\right)+a_{5}Y\left(t\right)\\ \;+a_{6}M\left(t\right)+A\left(t\right)+\varepsilon \;(t), \end{array}$$

For this analysis, those neurons that have significant coefficients for RPE (or *upQc*) at either reward site (*p* < 0.025; alpha = 0.05 was corrected for multiple comparisons) were determined to be RPE- (or *upQc*-) responsive neurons.

#### Coefficient for partial determination (CPD)

CPD for RPE and updated chosen value was calculated as the following (Neter et al., 1996; Kim et al., 2009):

(9) CPD(X2) = [SSE(X1)−SSE(X1, X2)]/SSE(X1),

where SSE(*Xi*) is the sum of squared errors of a regression model containing a set of independent variables *Xi, X*1 included *C*(*t*), *QL*(*t*), and *QR*(*t*) along with behavioral variables [*L*(*t*), *Y*(*t*), and *M*(*t*)], and *X*2 was either RPE or updated chosen value. Thus, CPD is the fraction of variance in neuronal activity that is additionally explained by RPE or updated chosen value.

#### Onset time of upcoming choice signals

To determine the time of choice onset (first behavioral manifestation of the animal's choice), we first estimated the Y-position in which the animal's X-position begins to diverge (near the upper T-junction in Figure 1A) for each session based on visual inspection. We then aligned the animal's X-position data relative to the time when the animal reached this Y-position, and choice onset was defined as the time when the animal's X-positions during the left-choice and right-choice trials became significantly different (*t*-test, *p* < 0.05; **Figure 4A**). Thus, choice onset was determined separately for each behavioral session. We then plotted temporal profiles of choice signals (fractions of neurons significantly responsive to the animal's upcoming choice) relative to choice onset (**Figure 4A**). The onset time of upcoming choice signals was when choice signals became significant for the first time and remained that way >1 s following choice onset.

#### Analysis of local field potentials (LFPs)

LFPs were recorded through one channel of each tetrode. For the identification of SWR events, LFPs were filtered between 100 and 250 Hz. The amplitude for each LFP trace was determined by the Hilbert transform, averaged across tetrodes and then smoothed with a Gaussian kernel (σ = 4 ms). SWR events were defined as the time periods when the smoothed envelop exceeded a threshold of the mean plus 2.5 SD for at least 20 ms (Jackson et al., 2006). Twenty milliseconds were added to the beginning and end of each SWR event. SWR events were analyzed only when animal's head speed was <4 cm/s.

### Statistical analysis

Statistical significance of a regression coefficient was tested based on a *t*-test, and significance of the fraction of neurons for a given variable was tested with a binomial test. Strengths of neural signals (fractions of neurons coding a given variable) between CA3 and CA1 were compared with a χ^2^-test. All statistical tests were based on two-tailed tests. A *p* < 0.05 was used as the criterion for a significant statistical difference. Data are expressed as mean ± *SEM* unless noted otherwise.

## Results

### Rat's choice behavior

All rats showed biased choices toward the higher-reward-probability target after block transition, an effect that was well-captured by a reinforcement learning (RL) model (Huh et al., 2009) (Figure 1B). The rat's choice behavior during the steady state (trials after reaching >90% of the maximum value in each block in 7-trial moving average) was consistent with the generalized matching law (Baum, 1974; Figure 1C). A logistic regression analysis revealed that the animal's choice was influenced by past choice outcomes, with more recent choice outcomes having stronger effects (Figure 1D). These results show that the animals were capable of tracking changes in relative reward probabilities based on past choice outcomes and adjusted their choices accordingly.

### Neural activity related to choice and reward

Units were recorded first from CA1 and then from CA3. All rats were over-trained in the task, and no significant difference was found in rat's choice behavior between CA1 and CA3 unit recording sessions (Figures 1C–E). In the present study, we focused our analysis on neural spike data at the reward sites to examine neural activity related to the evaluation of choice outcome. Neural spike data at the two reward sites were combined and analyzed together using multiple regression models so that neural activity related to chosen value (value of the chosen target in each trial) and action value (value of the left or right target) can be dissociated. Similar conclusions were obtained, however, when neural activity in each reward site was analyzed separately (see below).

**Figure 3:**
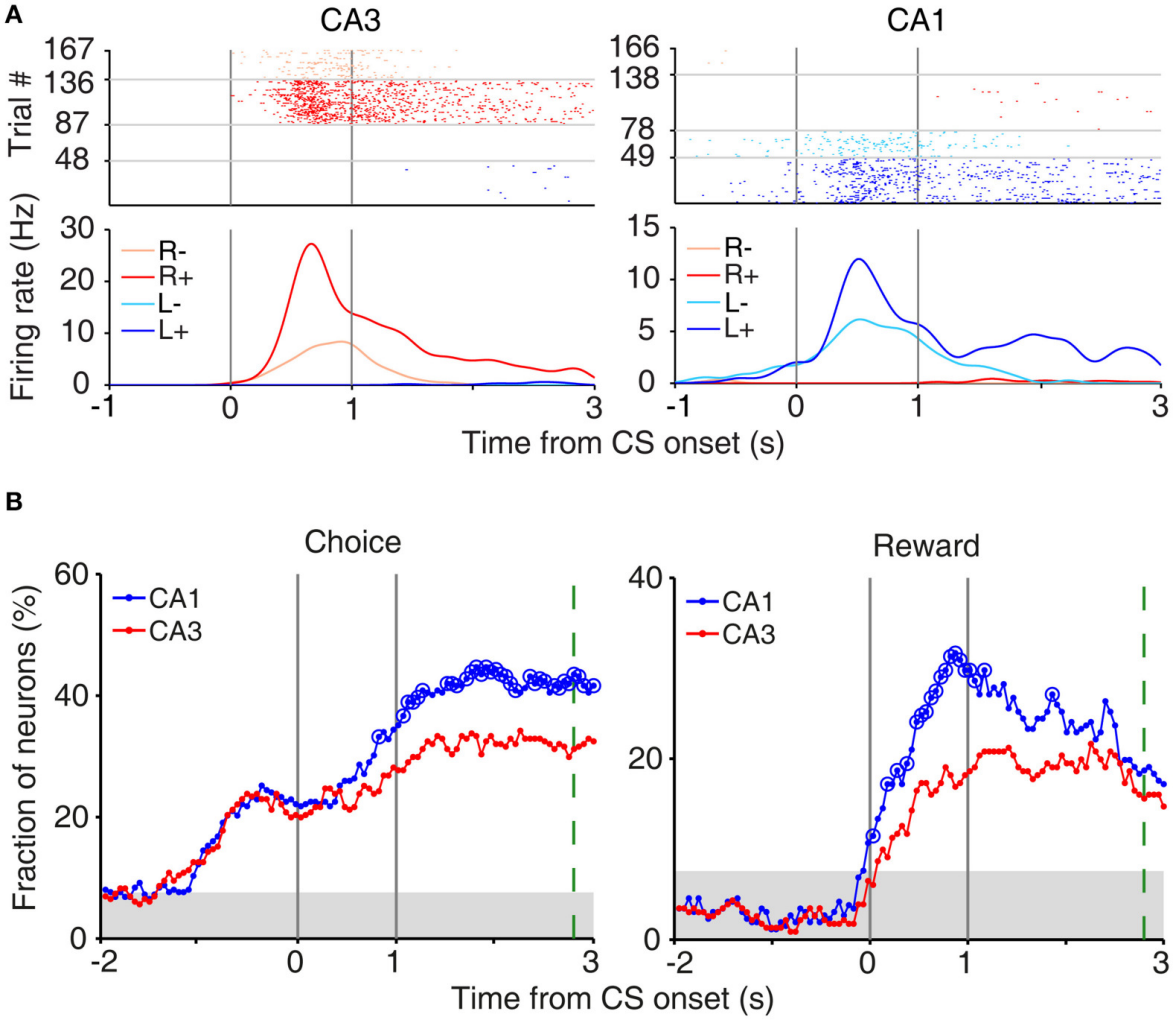
Neural activity related to choice and reward at reward sites. **(A)** Examples of choice- and reward-coding neurons at reward sites. Spike raster plots and spike density functions (σ = 100 ms) are shown for two example neurons that were responsive to both choice [left (L) vs. right (R) reward sites] and reward [reward (+) vs. no reward (−)]. Trials were grouped according to the animal's choice and reward. **(B)** Temporal profiles of choice and reward signals at reward sites, measured as the fraction of neurons that are significantly responsive to each variable (1 s moving window, 50 ms time steps). Large circles, significant differences between CA1 and CA3 (χ^2^-test, *p* < 0.05). Shading, chance level (binomial test). The green dashed line indicates the averaged time of reward stage offset in unrewarded trials.

In our task, the arrival of the rat at either goal site (breaking a photobeam sensor; Figure 1A) triggered an auditory tone (CS; 9 and 1 KHz in rewarded and unrewarded trials, respectively) signaling the availability of reward for 1 s before actual delivery of reward. This was to examine trial outcome-dependent neural activity while minimizing potential motor/sensory confounds; the animal's motor behavior and sensory inputs might differ between rewarded and unrewarded trials after actual delivery (or no delivery) of reward. We examined neural activity related to the rat's choice (*C*) and its outcome (or reward; *R*) at the reward site (between 2 s before and 3 s after CS onset) using a multiple regression model (Equation 3). Many CA3 and CA1 neurons were responsive to the rat's choice (left vs. right) and/or its outcome (reward vs. no reward) at the reward site (Figure 3A). Note that “choice”-related neural activity at reward sites merely represents side specificity of unit firing (i.e., place-specific firing). Temporal profiles of choice and reward signals (in terms of the fraction of neurons responsive to each variable; Equation 3) are shown in Figure 3B. As shown, both CA1 and CA3 conveyed strong neural signals for choice and reward when the choice outcome was revealed at the reward sites, with both choice and reward signals stronger in CA1 than CA3.

**Figure 4:**
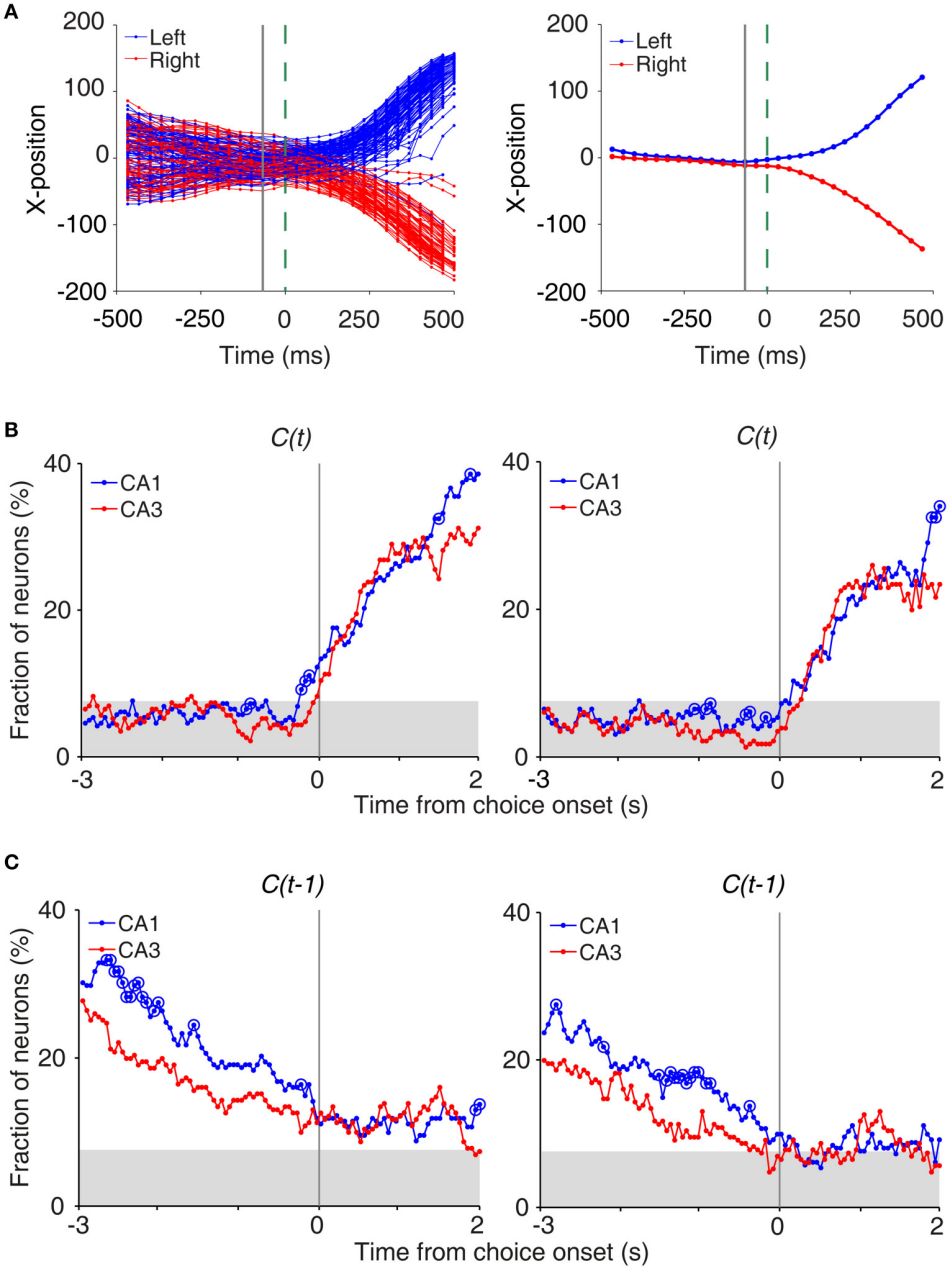
Neural activity related to upcoming and previous choices on central stem. **(A)** Determination of the time of choice onset. Choice onset was when the difference in the animal's horizontal coordinate (X-position) of the left- and right-choice trials first became statistically significant. Shown are the time course of the animal's X-position data near the upper T-junction of the maze (Figure 1A) during an example session (left, individual trials; right, mean). Blue and red indicate trials associated with the left and right goal choices, respectively. The dashed line (0 ms) indicates the time of clear separation in the animal's X-positions according to its choice upon visual inspection, and the solid line corresponds to the time when the difference in the X-positions for the left- and right-choice trials first became statistically significant (i.e., choice onset; *t*-test, *p* < 0.05) within ±0.5 s window from time 0. **(B)** Choice signals around choice onset examined with 1 s (left) and 0.5 s (right) moving windows (50 ms time steps). **(C)** Previous choice signals examined with 1 s (left) and 0.5 s (right) moving windows (50 ms time steps). The same format as in Figure 3B.

We also examined whether CA3 or CA1 neurons conveyed information on the rat's upcoming choice (Frank et al., 2000; Wood et al., 2000; Ito et al., 2015) when the animal was on the central stem of the maze (Equation 3). For this analysis, we aligned neural activity to the onset of choice behavior (the first time point for behavioral manifestation of the rat's upcoming target choice; Figure 4A) that was determined based on the animal's movement trajectories in each behavioral session as previously described (Kim et al., 2009, 2013; Sul et al., 2010, 2011). A sliding window analysis (1 s window advanced in 50 ms steps) showed that choice signals were weak before behavioral manifestation of the rat's goal choice in both CA3 and CA1 (Figure 4B). An analysis at a higher temporal resolution (0.5 s moving window) showed that significant choice signals were evident in both CA1 and CA3 only after behavioral manifestation of the animal's choice (Figure 4B). The current task allows separate examinations of neural activities related to previous and future choices, because they were only modestly correlated (*r* = 0.037 ± 0.154; mean ± *SD* across sessions). Both CA1 and CA3 carried relatively strong previous choice signals on the central stem of the maze (Equation 3; Figure 4C), which is consistent with our previous finding (Lee et al., 2012). Thus, both CA1 and CA3 conveyed strong retrospective choice signals, but weak prospective choice signals, on the central stem of the maze.

### Neural activity related to chosen value

**Figure 5:**
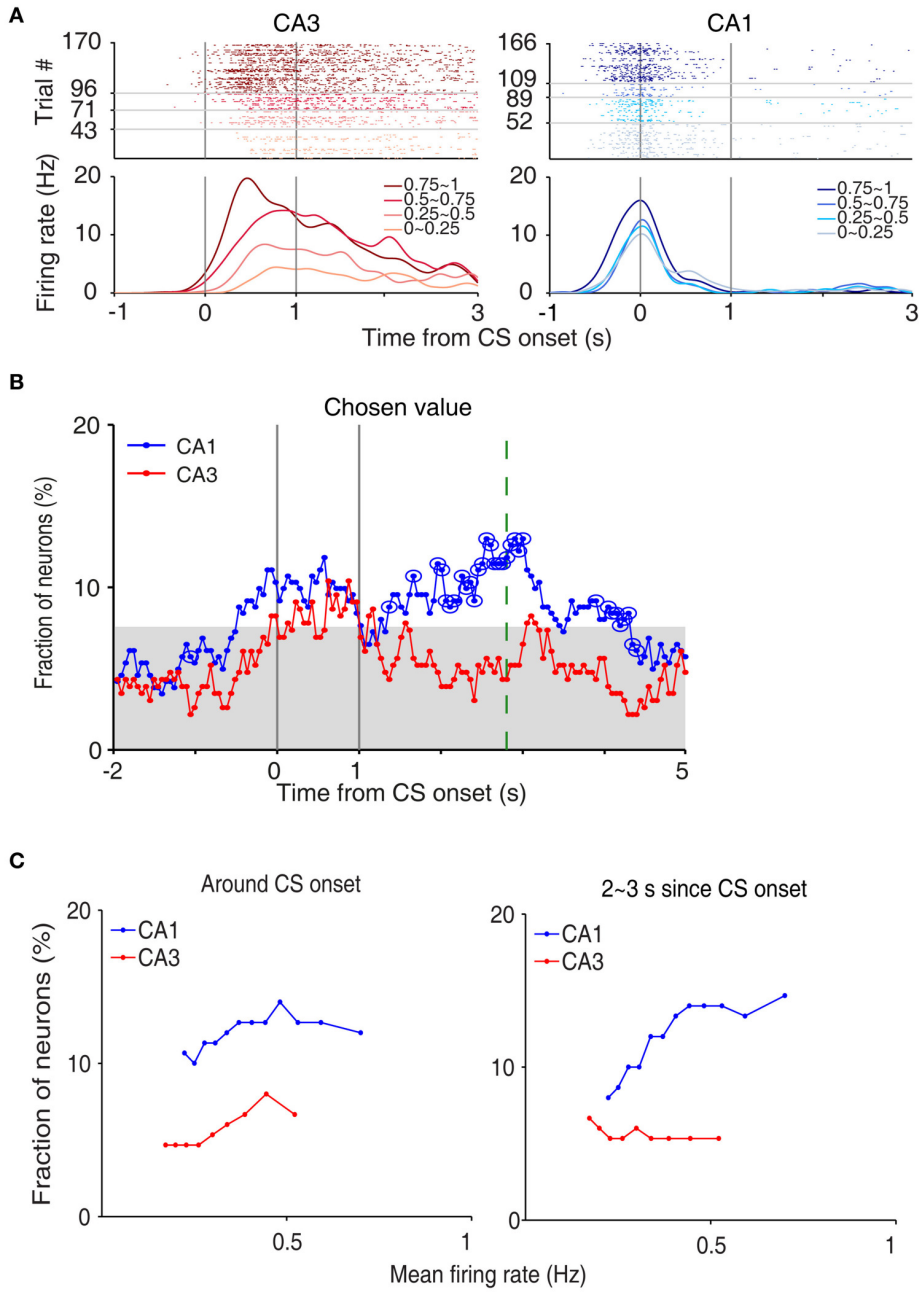
Neural activity related to chosen value. **(A)** Two examples of chosen value-coding neurons. Trials were grouped according to chosen value (in quartiles). **(B)** Temporal profiles of chosen value signals. The same format as in Figure 3B. **(C)** Chosen-value signals as a function of firing rate (mean of 150 neurons, steps of 10 neurons). CA3 and CA1 units were aligned according to their mean discharge rates, and chosen value signals around CS onset (2 s window centered on CS onset) and during 2–3 s since CS onset were examined using a moving window of 150 neurons that was advanced in steps of 10 neurons (starting from the low-firing side).

**Figure 6:**
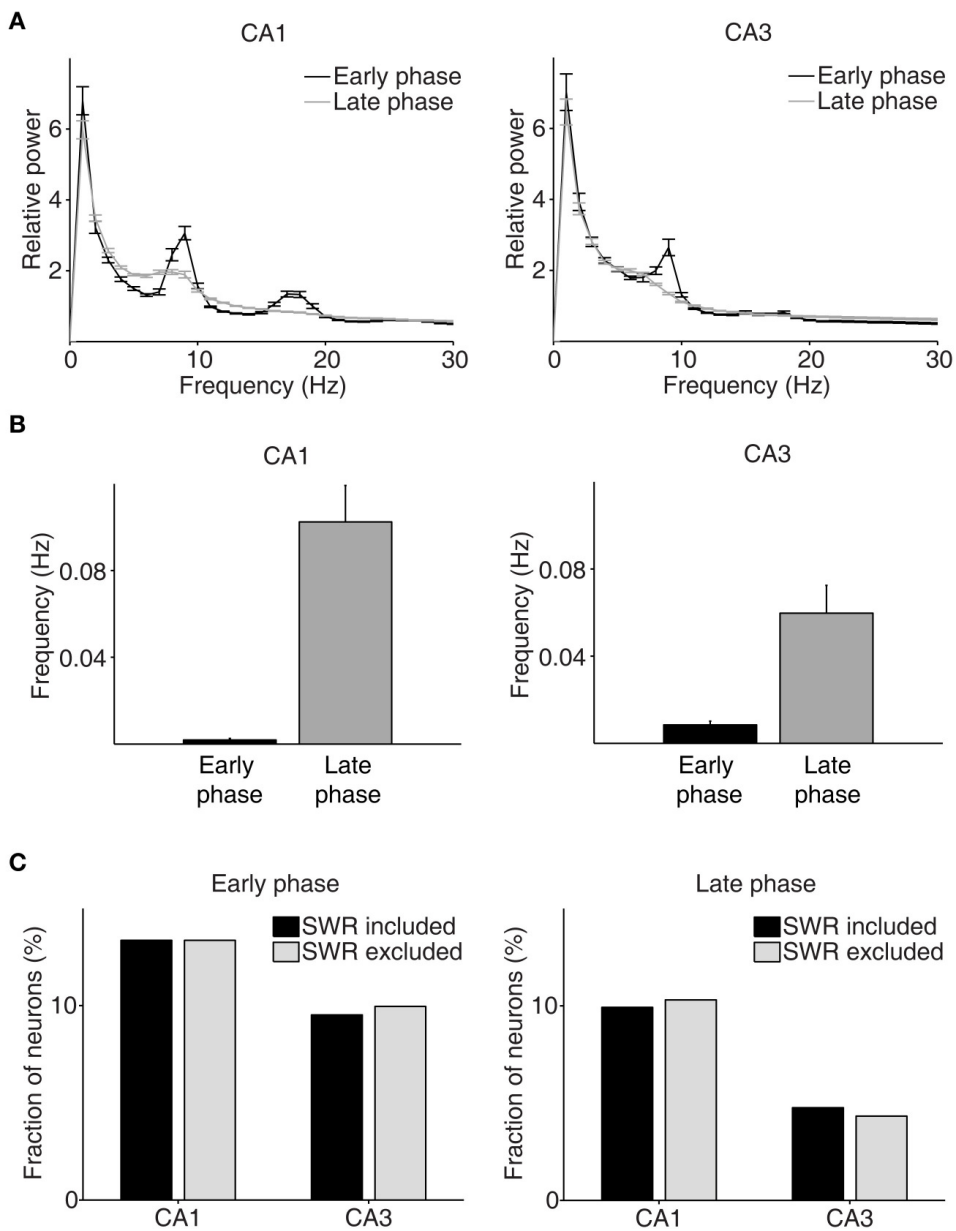
Results of LFP analysis. **(A)** LFP power spectrum during the early (between –1 and 1 s since CS onset) and late (between 2 s since CS onset and exit from the reward site) phases of the outcome period (CA1, *n* = 32 sessions; CA3, *n* = 49 sessions). **(B)** The frequency of SWRs (events per second) during the early and late phases of the outcome period. **(C)** Chosen value signals, that were estimated with and without excluding the neural data associated with SWRs, were compared.

We then examined neural activity related to chosen value (value of the chosen target in each trial), that was estimated with the SP model, using a multiple regression model (Equation 4; Figure 5A). As shown in our previous study (Lee et al., 2012), chosen value signals began to rise ~1 s before CS onset in CA1, indicating that expected reward signals emerged in CA1 before choice outcome was revealed. CA1 chosen value signals stayed well-above chance level during the CS period and then subsided below chance level at CS offset. Thus, neural signals necessary to update value of the chosen action, namely reward and chosen value signals, coexisted during the CS period in CA1, which is consistent with our previous findings (Lee et al., 2012). Chosen value signals were also elevated in CA3 when the outcome of the animal's choice was revealed at the reward sites. However, compared to CA1, CA3 chosen-value signals rose above chance level more slowly (500 and 100 ms before outcome onset for CA1 and CA3, respectively; Figure 5B), and were significantly weaker (CA3, 8.2%; CA1, 14.1%; χ^2^-test, *p* = 0.040) when analyzed using a relatively large analysis time window (between −0.5 and +1 s relative to outcome onset). Moreover, after subsiding at ~1 s following outcome onset, chosen-value signals rose again in CA1, but not in CA3 (Figure 5B). Chosen value signals around CS onset (2 s window centered on CS onset) and during late reward period (2–3 s since CS onset) were consistently stronger in CA1 than CA3, as shown by a plot of strength of chosen-value signals as a function of mean discharge rate during the task (Figure 5C). Sharp-wave ripple (SWR) events were rare at reward sites in our study; consequently, similar levels of chosen-value signals were found after excluding neural activity associated with SWR events (Figure 6), indicating that our results are independent of reward-enhanced, SWR-associated replays of place cell activity (Foster and Wilson, 2006; Singer and Frank, 2009; Pfeiffer and Foster, 2013; Ólafsdóttir et al., 2015).

### Neural activity related to reward prediction error and updated chosen value

**Figure 7:**
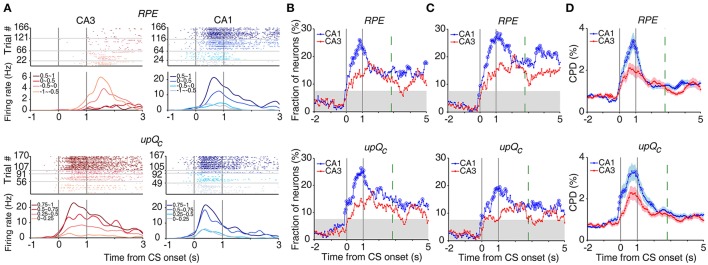
Neural activity related to RPE and updated chosen value. **(A)** Example neurons coding RPE (top) or updated chosen value (*upQc*; bottom). Trials were grouped according to RPE or updated chosen value (in quartiles). **(B)** Temporal profiles of RPE and updated chosen value signals. The same format as in Figure 3B. **(C)** Neural activity at each goal site was analyzed separately. **(D)** CPD for RPE and updated chosen value. Shading indicates SEM.

Chosen value and outcome signals can be combined to compute RPE and update chosen value (Kim et al., 2009; Sul et al., 2010, 2011; Lee et al., 2012). Both RPE and *upQc* signals, that were examined with multiple regression models (Equations 5 and 6), were stronger in CA1 than CA3 (Figures 7A,B). An analysis of neural activity separately at each goal site (Equations 7 and 8) yielded similar results (Figure 7C). An analysis examining the effect sizes of all neurons, rather than the fraction of significant neurons, using CPD (Equation 9) also yielded similar results (Figure 7D).

## Discussion

We have shown previously that CA1 conveys neural signals necessary to update value of the chosen target in a dynamic foraging task (Lee et al., 2012). In the present study, we compared neuronal activity related to updating the value of chosen target in the CA3 vs. CA1 regions in the identical behavioral task. We replicated our previous findings in the present study; reward and chosen value signals converge in CA1 when trial outcome was revealed. In addition, we found that CA3 also conveys significant reward and chosen value signals when trial outcome was revealed. However, reward and chosen value signals were stronger in CA1 than CA3 and, consequently, signals for RPE and updated chosen value were also stronger in CA1 than CA3. These results are in line with a finding that spatial firing of CA1, but not CA3, neurons is reorganized to represent new goal locations (Dupret et al., 2010). They are also consistent with our recent finding that selective inactivation of CA1, but not CA3, impairs value learning (Jeong et al., 2016). Together with our previous finding that value signals are stronger in CA1 than the subiculum (Lee et al., 2012), these results raise the possibility that CA1 might play a particularly important role among hippocampal subregions in evaluating experienced events.

Our results indicate that at least some of CA1 value signals are independent of value-dependent discharges of CA3 neurons. What would be the neural basis of CA3-independent value signals in CA1? One possibility would be differential effects of dopamine on CA3 and CA1 neural activity. Dopaminergic projections from the ventral tegmental area (VTA) and dopamine receptor subtype distributions are different between CA3 and CA1 (Gasbarri et al., 1997; Shohamy and Adcock, 2010; c.f., Takeuchi et al., 2016). Dopamine conveys RPE signals (Schultz et al., 1997; Roesch et al., 2007; Cohen et al., 2012) and modulates synaptic transmission/plasticity in CA1 (e.g., Frey and Schroeder, 1990; Otmakhova and Lisman, 1996; Li et al., 2003; O'carroll and Morris, 2004; Zhang et al., 2009; Hansen and Manahan-Vaughan, 2012; Brzosko et al., 2015; Rosen et al., 2015). Dopamine might differentially affect CA3 vs. CA1 neurons through these mechanisms so that CA1 neuronal activity is modulated by value independent of CA3 inputs. This possibility is supported by the finding that inactivation of the ventral tegmental area affects spatial firing of CA1, but not CA3, place cells (Martig and Mizumori, 2011). It is also possible that other afferent projections to CA1, such as direct layer III entorhinal cortical projections (Witter, 1986, 1993; Amaral, 1993), CA2 projections (Tamamaki et al., 1988; Shinohara et al., 2012; Kohara et al., 2014), prefrontal cortical projections (Rajasethupathy et al., 2015), and thalamic projections (Herkenham, 1978; Wouterlood et al., 1990), contribute to value-related neural activity of CA1 neurons. Future studies combining manipulation of specific afferent projections and monitoring CA1 neural activity would be helpful in elucidating roles of dopaminergic and other afferent projections in CA1 value processing.

Recent studies indicate an important role of the hippocampus in imagining future episodes (Buckner, 2010; Schacter et al., 2012; Gaesser et al., 2013; Mullally and Maguire, 2014). In rats, hippocampal place cells go through sequential discharges (replays) during sleep and awake immobility that reflect experienced as well as unexperienced trajectories (e.g., Louie and Wilson, 2001; Lee and Wilson, 2002; Diba and Buzsáki, 2007; Johnson and Redish, 2007; Gupta et al., 2010; Carr et al., 2011; Dragoi and Tonegawa, 2011; Pfeiffer and Foster, 2013). Our results suggest that replay of CA1 place cells may be affected by value information represented in CA1. Consistent with this possibility, trajectories reconstructed from replays of CA1 place cells are preferentially directed to previously visited as well as unvisited (but observed) reward locations in rats (Foster and Wilson, 2006; Pfeiffer and Foster, 2013; Ólafsdóttir et al., 2015). Replay of place cells involving value-coding CA1 neurons might be a way of evaluating expected values of replayed place cell sequences, which would be useful for simulating the most probable and rewarding trajectories (or event sequences) for maximizing value. Additional studies are needed to explore whether and how value-dependent firing of CA1 neurons contributes to the evaluation of simulated trajectories.

## Author contributions

SL and MJ conceived the study. SL, NH and JL performed the experiments. SL, NH, JL, JG, and MJ analyzed the data; and IL and MJ wrote the paper with inputs from all authors.

### Conflict of interest statement

The authors declare that the research was conducted in the absence of any commercial or financial relationships that could be construed as a potential conflict of interest.
